# Supplementing Trace Minerals to Beef Cows during Gestation to Enhance Productive and Health Responses of the Offspring

**DOI:** 10.3390/ani11041159

**Published:** 2021-04-18

**Authors:** Kelsey Margaret Harvey, Reinaldo Fernandes Cooke, Rodrigo da Silva Marques

**Affiliations:** 1 Prairie Research Unit, Mississippi State University, Prairie, MS 39756, USA; kelsey.harvey@msstate.edu; 2 Department of Animal Science, Texas A&M University, College Station, TX 77843, USA; 3 Department of Animal and Range Sciences, Montana State University, Bozeman, MT 59717, USA; rodrigo.marques@montana.edu

**Keywords:** beef cows, developmental programming, gestation, health, offspring, production, trace minerals

## Abstract

**Simple Summary:**

During gestation, the fetus relies on the dam for the supply of all nutrients, including trace minerals, which are essential for developmental processes including organogenesis, vascularization, and differentiation. Alterations in maternal nutritional status may promote adaptations that permanently alter the trajectory of growth, physiology, and metabolism of the offspring. Supplementing trace minerals to gestating cows may be a strategy to enhance progeny performance and health. The purpose of this review is to highlight current information relevant to trace mineral supplementation during gestation, with an emphasis on Zn, Cu, Co, and Mn, and their impacts on offspring productive responses. Identifying nutritional strategies targeted at this period of development and understanding the implications of such provides an opportunity to enhance the productive efficiency of beef cattle systems.

**Abstract:**

Nutritional management during gestation is critical to optimize the efficiency and profitability of beef production systems. Given the essentiality of trace minerals to fetal developmental processes, their supplementation represents one approach to optimize offspring productivity. Our research group investigated the impacts of supplementing gestating beef cows with organic-complexed (AAC) or inorganic sources (INR) of Co, Cu, Mn, or Zn on productive and health responses of the progeny. Calves born to AAC supplemented cows had reduced incidence of bovine respiratory disease and were >20 kg heavier from weaning until slaughter compared to unsupplemented cohorts. Complementing these findings, heifer progeny born to AAC supplemented cows had accelerated puberty attainment. Collectively, research demonstrates supplementing trace minerals to gestating beef cows may be a strategy to enhance offspring productivity in beef production systems.

## 1. Introduction

Over the course of the next 30 years, the global population is expected to reach over 9.1 billion people, and as a result, food production will need to increase by over 70% [1]. Of the total increase in food production required, meat production will need to rise by approximately 200 million tonnes and accordingly, there will be greater demand for beef products [1]. Concomitantly, urban areas will expand, reducing natural resources and land available for beef cattle production and other agricultural activities. Hence, advancements in both management and technologies are required to reach a level of animal production that can provide for the population by 2050 [1].

Cow-calf production systems are the foundation for the US and worldwide beef industries by determining the number of cattle available for harvest. The success of each cow-calf operation depends on its ability to produce one healthy calf per cow annually, and cow-calf producers are constantly challenged to improve growth, efficiency, carcass, muscle, and quality characteristics of offspring [2]. Nutritional manipulation during periods of developmental plasticity such as the embryonic, fetal, and neonatal periods exerts short- and long-term consequences on skeletal muscle and adipose tissue development, health, and overall performance of offspring [3,4]. Improved skeletal muscle development and adipogenesis in skeletal muscle enhance growth performance and carcass marbling upon slaughter [5], traits that benefit carcass value and beef palatability [6]. Moreover, the development of replacement beef heifers is a critical component of cow-calf systems, and production efficiency increases with improved female longevity [7]. Maternal nutrient status during fetal development also affects the lifetime productivity of female progeny by impacting ovarian reserves [8,9]. Identifying specific nutritional strategies that are targeted at these critical periods of development provides a unique opportunity to optimize the efficiency and profitability of beef cattle systems. Hence, the purpose of this review is to highlight current information relevant to trace mineral supplementation to beef cows during gestation, with an emphasis on Zn, Cu, Co, and Mn, and their impacts on offspring productive responses.

## 2. Developmental Programming

Developmental programming, also called fetal programming, proposes that alterations in fetal nutritional or endocrine status may result in adaptations that permanently alter the trajectory of growth, physiology, and metabolism of the offspring [3,10]. Moreover, recent reviews focused on developmental programming have suggested that nutritional manipulation during gestation can have profound influences on the occurrences of adulthood disease and long-term performance [3,11], given that development of the fetus is sensitive to direct and indirect effects of maternal nutrition at all stages between oocyte maturation to parturition [12,13]. Hence, effective prenatal nutritional management represents an opportunity to alter offspring development and performance.

The fetal stage is critical for skeletal muscle and intramuscular adipocyte development [14,15], whereas a reduction in the number of muscle fibers permanently reduces animal performance, and adipocyte development provides sites for marbling formation during the feedlot finishing period [5]. Maternal nutrient restriction results in a reduction in the total number of secondary muscle fibers [14], however, maternal overnutrition results in elevated expression of adipogenic genes in fetal skeletal muscle [16] and increased number and size of adipocytes in the skeletal muscle postnatally [17]. The effect of maternal nutrient status during gestation can also have considerable impacts on the future reproductive performance of female progeny. For heifers to be efficient, they need to attain puberty by 12 months of age and conceive early in their first breeding season [18]. Therefore, nutritional management to maximize the number of replacement heifers pubertal by 12 months of age are critical to the productivity of cow-calf operations. For example, heifers born to protein supplemented dams had greater conception rates during the first 21 days of the breeding season [19]. Heifers born to energy-restricted dams had a reduction in antral follicle count compared to heifers born to control-fed cohorts [9,20,21], a trait that is positively correlated with pregnancy success in both beef heifers and dairy cows [22,23,24]. Suboptimal maternal nutrient status during gestation also results in alterations within the neuroendocrine system of the female offspring, leading to delayed puberty attainment and disrupted reproductive function [25]. Taken together, these findings indicate maternal nutritional status during gestation significantly impacts offspring growth and development [3], leading to long-term effects on performance and quality of carcass traits [3,26] and altered reproductive function of female progeny [27].

### 2.1. Dietary Factors and Developmental Programming

Maternal macronutrient nutrition has been reviewed several times in the literature [3,13,28], but little is known about the effects of maternal trace mineral supplementation on progeny health and performance. For example, protein supplementation provided to cows during late gestation has been shown to increase offspring birth and weaning weights [29], increase the postweaning rate of bodyweight gain [19], and enhance carcass marbling [26]. Increasing dietary energy during late gestation results in increased progeny birth body weight [30], whereas energy source during gestation also affects fetal growth. Research has demonstrated offspring from cows supplemented with corn or dried distillers’ grains with solubles were heavier at birth and tended to be heavier at weaning compared with offspring from cows fed grass hay at isocaloric intake [31]. Other nutrients, including trace minerals, are essential for fetal developmental processes including protein synthesis, bone formation, and lipid metabolism [32], and warranted investigation.

### 2.2. Trace Minerals

Cow-calf production systems depend on forages as the main source of nutrients to cattle, which may be variable in quality and might not be nutritionally complete [33]. Forages provide minerals that are vital for nearly all processes within the body. Many factors affect the mineral content of forages including soil, plant species, stage of maturity, yield, climate, and management practices [33]. However, forages often do not provide enough minerals to meet the requirements of livestock [34]. Hence, cow-calf producers must design and integrate mineral supplementation programs according to animal requirements and forage characteristics to maintain maximum long-term productivity [35]. Minerals are divided into two classes; macro minerals which are required in amounts > 100 ppm of the diet, and trace minerals which are required in amounts < 100 ppm of the diet and do not change with animal stage or level of production [36]. Although trace minerals such as Cu, Zn, I, Mn, Se, Co, and Fe are present in only minute amounts, they are essential for beef cattle homeostasis [36]. In fact, trace mineral deficiencies during fetal life are known to persistently affect the growth, immunological, and morphological development of a variety of fetal and neonatal tissues [37] given that these elements play numerous roles in enzymatic and metabolic functions during fetal development [32]. Still, the fetus relies fully on the maternal–fetal interface for a proper supply of trace minerals [38]. For example, Gooneratne and Christensen [39] reported that fetal liver Cu concentrations are greater than that of the maternal liver, suggesting that the maternal system shunts Cu to support fetal development [39]. Hansard [40] investigated the placental transfer of radioactive Mn from maternal to fetal tissue and noted that only 15% of the radioactive Mn was absorbed by the cows and 70% of the Mn was deposited in fetal tissue. Hence, these results indicate that fetal requirements for trace minerals take priority over maternal requirements for these elements throughout gestation.

### 2.3. Inorganic vs. Organic Trace Mineral Supplementation

Trace minerals have been traditionally added to animal diets to meet requirements, however, the source of mineral supplementation may affect individual animal performance. Organic trace minerals differ from inorganic sources due to their chemical association with an organic ligand, as usually trace minerals are found as inorganic salts [41]. Recently, there has been marked interest in the use of organic trace minerals in ruminant diets due to indications that these may be of greater bioavailability compared to their inorganic counterparts [42]. Metal chelates, or complexes that constitute an organically bound trace mineral, are hypothesized to be stable in the digestive tract, thus protecting the metal from forming complexes with other dietary components that would inhibit absorption, and perhaps allowing greater absorption of the trace mineral [41]. In order for an animal to utilize inorganic trace minerals, the animal must first convert them to organic biologically active forms, given that trace minerals in the body function almost entirely as organic complexes or chelates already [41]. Therefore, ruminants may respond to supplementation in the form of enhanced growth, milk production, reproduction, and immune responses. 

Supporting this rationale, Nocek et al. [43] reported that cows can be supplemented with organic trace minerals at 75% of requirements with no reduction in reproductive or productive performance compared with supplementing 100% of requirements using only sulfate sources. Organic trace mineral supplementation to dairy cows significantly increased milk production, milk fat yield, and milk protein yield compared to inorganic trace mineral supplemented cohorts [44]. Previous research has demonstrated supplemented organic complexed trace minerals improved reproductive performance in cattle, decreasing interval to first estrus and increasing pregnancy rates [45,46], as well as resulting in improved in vitro embryo production efficiency [47]. A meta-analysis by Rabiee et al. [48] reported that lactating dairy cows supplemented with organic-complexed trace minerals have greater milk yield, fewer days open, and require fewer services per conception compared with inorganic supplemented cohorts. Ahola et al. [49] also reported an improved pregnancy rate to AI of grazing beef cows supplemented with organic-complexed trace minerals compared with non-supplemented cohorts. Collectively, these results demonstrate organic-complexed trace mineral supplementation positively impacts cow productive and reproductive responses.

Supplying organic complexed trace minerals has also been shown to enhance animal productivity and immune parameters during challenging stages such as the periparturient period [50,51], perhaps due to the participation of trace elements in enzymatic and antioxidant systems [52,53] that may have major implications to future progeny performance. In fact, organic Cu, Mn, and Zn supplementation during late gestation improved calf serum antioxidant capacity and immunoglobulin concentrations compared with calves from non-supplemented cows [54]. Formigoni et al. [55] supplemented inorganic or organic Zn, Mn, and Cu to gestating Holstein cows and noted an increased concentration of immunoglobulins in the colostrum and reduced calf mortality in cows fed organic trace minerals compared with inorganic-supplemented cohorts. Nonetheless, research investigating the impacts of trace mineral source supplementation on beef cattle performance and health has been variable, potentially due to interacting factors present across experiments including, but not limited to, trace mineral antagonists, environmental pressures, duration of supplementation, and maternal trace mineral status [33,49,56].

## 3. Supplementing Organic-Complexed Trace Minerals during Late-Gestation

The fetus depends on the dam for the proper supply of all nutrients during gestation, including trace minerals [38]. Further, if maternal supply is inadequate or supply to the fetus is impaired, developmental processes and postnatal performance may be hindered [57]. Trace elements such as Zn, Cu, Mn, and Co are required for the proper development of fetal nervous, reproductive, and immune systems [32,58], underscoring the importance of supplying these nutrients to gestating beef cattle. Hostetler et al. [32] reported supplementing an organic Cu, Mn, and Zn to gestating sows increased concentrations of these trace minerals in fetal tissues and reduced fetal loss by d 30 of gestation. Supporting this rationale, our research group reported that supplementing late gestating beef cows with an organic complexed source of Co, Cu, Zn, and Mn instead of no supplementation optimized offspring productivity [59]. This initial project was conducted with beef cows supplemented with sulfate sources of Cu, Co, Mn, and Zn (INR), organic complexed Cu, Co, Mn, and Zn (AAC), or no supplemental Cu, Co, Mn, and Zn. Marques et al. [59] conducted this trial using cows at the end of their second trimester of gestation (d 0 of the experiment), and formulated all diets to meet the requirements for energy, protein, Se, I, and vitamins of pregnant cows during the last trimester of gestation [60]. The INR and AAC sources were formulated to provide the same daily amount of Cu, Co, Mn, and Zn. As expected, supplementing INR or AAC increased cow liver concentration of Co, Cu, and Zn in samples collected prior to calving (d 75) compared with non-supplemented cohorts (Table 1). Marques et al. [59] also reported liver Cu and Zn concentrations in the neonatal calf were only increased in calves born to AAC supplemented cows compared with non-supplemented cows (Table 1). As stated previously, organic trace minerals are expected to have enhanced absorption, retention, and overall bioavailability [32,41], however only cow liver Co concentrations supported this rationale. Corroborating the results of Marques et al. [59] others have also reported variable effects of supplementing organic Zn, Cu, and Co on the liver mineral status of beef cattle [49,61,62].

The current requirements for trace minerals were established to support normal growth, reproductive, and immune functions but appear to be insufficient when cattle are exposed to stressors from management practices, such as weaning, road transport, and arrival at the feedlot [63]. Given the essentiality of Cu, Mn, Co, and Zn for proper development of the fetal immune system [31,48] and their profound effects on antioxidant and enzyme components [64,65], maternal dietary trace mineral supply might be one feasible nutritional alternative to program the immune system of the fetus. Accordingly, Jacometo et al. [66] reported that calves born from cows supplemented with organic trace minerals during late gestation had decreased expression of markers of inflammation and oxidative stress at 3 weeks of age compared to calves born to inorganic supplemented cows. These authors hypothesized that maternal supplementation with organic trace minerals, may alter the immune response of the neonate, but noted that further research was needed to determine if the effects would persist [66]. In turn, Marques et al. [59] reported that calves born to AAC cows had a reduced BRD incidence compared with calves from non-supplemented cows (Table 1), suggesting that feeding the AAC diet to late gestating beef cows resulted in programming effects on postnatal offspring health.

As previously mentioned, maternal nutrient inputs during gestation have a large impact on biological mechanisms involved in fetal growth and nutrient utilization that may affect progeny performance and health later in life [67,68]. The fetal growth trajectory is persistently affected by maternal nutrient intake from the early stages of fetal development to birth, whereas the majority of research is directed toward the last trimester of gestation when approximately 75% of the fetal growth occurs and nutrient requirements for fetal growth are maximal [36,68]. Trace minerals, for example, are involved in all stages of cell growth and differentiation and are vital components of many enzymes and cell structures [69]. These elements affect fetal development by altering hormones, growth factors, and cell signaling pathways involved in nutrient uptake by the fetus, which may irreversibly impact progeny productivity [3,69,70]. Although no treatment differences were detected for calf birth body weight (BW), Marques et al. [59] reported that organic Cu, Co, Mn, and Zn supplementation to beef cows during late-gestation increased BW of their offspring by 24 kg at weaning and by 31 kg at slaughter compared with calves from non-supplemented cows (Table 1). Others investigating trace mineral supply in maternal diets have also demonstrated changes in progeny performance trajectory [49,61]. Stanton et al. [61] noted that supplementing late-gestating beef cows with organic-complexed Cu, Zn, Co, and Mn improved progeny weaning weight compared with calves from cows that received sulfate sources. Collectively, the results from Marques et al. [59] were novel, and suggestive of programming effects of organic trace minerals on postnatal offspring performance and health [13]. Nevertheless, Marques et al. [59] recognized that the physiological mechanisms underlying these outcomes were still unknown and warranted investigation.

## 4. Supplementing Organic-Complexed Trace Minerals Mid- to Late-Gestation

Nutritional management of beef cows impacts fetal development throughout the entirety of gestation [3], whereas Marques et al. [59] investigated supplementation during the last trimester of gestation. Supplementing organic trace minerals may be of even greater benefit if offered to beef cows over a greater duration of gestation. Moreover, Marques et al. [59] did not evaluate the potential physiological mechanisms by which organic trace mineral supplementation positively impacted postnatal offspring performance and health [59]. A majority of U.S. cow-calf herds receive supplementation [71] underscoring the importance of deciphering the difference between organic complexed and sulfate sources of trace minerals, without the inclusion of a non-supplemented group. Therefore, the next step was to evaluate the effects of supplemental organic complexed or sulfate sources of Co, Cu, Mn, and Zn to beef cows during the second and third trimesters of gestation. 

In Harvey et al. [72], non-lactating pregnant beef cows were supplemented with sulfate sources of Cu, Co, Mn, and Zn (INR) or organic complexed Cu, Co, Mn, and Zn (AAC) beginning at the end of the first trimester of gestation (d 0 of the experiment). The INR and AAC diets were formulated to provide the same daily amount of energy, protein, macro minerals, and trace minerals including Co, Cu, Mn, and Zn [59]. In samples collected during late gestation (d 97), Harvey et al. [72] reported greater liver Co, less liver Cu, and similar liver Zn and Mn between cows supplemented with sulfate or organic complexed sources (Table 2), corroborating results reported by Marques et al. [59]. However, Harvey et al. [72] reported greater liver Cu and Zn concentrations in cows receiving INR diets compared to AAC cohorts upon calving, whereas no treatment differences were detected for liver Co or Mn (Table 2). These latter outcomes do not corroborate that organic trace mineral sources have increased absorption and retention compared with sulfate mineral sources [32,41]. As noted by Harvey et al. [72], liver concentrations are often used as the standard for assessing mineral status in livestock, however, they should not be used as an absolute indicator of trace mineral status [73]. In turn, Harvey et al. [72] reported greater hepatic mRNA expression of metallothionein 1A (MT) in AAC supplemented cows, and similar hepatic mRNA expression of Cu-transporter protein (CUT), and Cu-Zn-superoxide dismutase 1 (SOD) compared with INR cows at calving (Table 2). These genes are associated with Cu and Zn metabolism in the liver and provide additional insight into the Zn and Cu status of cattle [74,75,76,77]. Hence, these outcomes suggest that Cu and Zn status between AAC and INR cows may not have differed during gestation and upon calving as denoted by differences observed in liver concentrations of these trace minerals.

To complement and provide further biological support to Marques et al. [59], Harvey et al. [72] also examined trace mineral concentrations in placental cotyledons and calf liver at birth and 24 h after birth. These authors, however, reported no differences in trace mineral profile of placental and calf liver samples from AAC-supplemented cows or those receiving INR diets (Table 3). Supplementing gestating beef cows with AAC also did not impact hepatic mRNA expression of CUT, MT, or SOD in calves at birth or 24 h later (Table 3), further suggesting similar hepatic Cu and Zn metabolism during early life [75,77,78]. Harvey et al. [72] also reported liver concentrations of Cu, Co, Mn, and Zn decreased in all calves from birth to 24 h after birth (Table 3), which was expected given that the bovine neonate depends heavily on liver stores of trace minerals for postnatal utilization due to low concentrations of these elements in colostrum and milk [37]. Accordingly, hepatic mRNA expression of CUT and MT increased in all calves 24 h after birth (Table 3), again reflecting the greater activity in hepatic tissue and utilization of trace minerals [37,79].

In an effort to elucidate the physiological mechanisms by which providing the AAC diet to gestating beef cows stimulated offspring growth via programming effects reported by Marques et al. [59], Harvey et al. [72] evaluated mRNA expression of genes associated with adipogenic and myogenic activities in the *longissimus* muscle (LM) in calves at birth and at weaning. Supplementing gestating beef cows with Cu, Co, Mn, and Zn as organic complexed instead of sulfate sources did not alter mRNA expression of genes associated with these activities in the LM of calves at birth or upon weaning, despite the established role of trace minerals on muscle development and adipogenesis [80,81]. These outcomes resulted in similar offspring growth from birth until weaning (Table 4). Harvey et al. [72] also reported similar milk production and profile between AAC and INR cows (Table 4), indicating that any potential treatment effects on offspring responses were not caused by alterations in milk yield during early lactation [72]. Secretion of trace elements in the milk is strictly regulated within the mammary gland, avoiding imbalances even when trace minerals are supplied in excess of requirements [82], corroborating the lack of treatment differences reported by Harvey et al. [72] for lactation variables. Marques et al. [59] also did not report differences in offspring weaning responses between AAC and INR supplemented cows, despite a 13 kg numerical increase in weaning BW from AAC cows. Collectively, Harvey et al. [72] reported that supplementing Co, Cu, Zn, and Mn as organic complexed or sulfate sources yielded similar cow-calf productive responses until weaning.

### Impacts on Female Progeny Reared as Replacement Heifers

Both male and female offspring were reared for slaughter by Marques et al. [59], whereas maternal nutrition and weaning BW have tremendous impacts on female reproductive development [27,59], and research investigating trace mineral supplementation during gestation and female progeny performance is limited. For example, no differences in puberty attainment or pregnancy status were reported for heifers born to organically supplemented dams compared to inorganic supplemented cohorts [83]. However, Price et al. [83] began supplementation approximately 82 d prior to calving, well after ovarian development of female progeny was complete [84]. Therefore, Harvey et al. [85] aimed to evaluate the effects of supplementing organic complexed or sulfate sources of Co, Cu, Mn, and Zn to beef cows during the second and third trimesters of gestation on post-weaning responses of the female offspring reared as replacement heifers.

**Figure 1:**
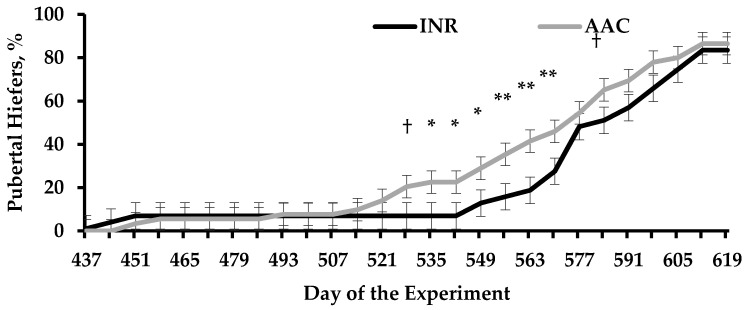
Puberty attainment of replacement heifers from beef cows that received diets containing supplemental sulfate sources of Co, Cu, Mn, and Zn (INR) or organic-complexed source of Co, Cu, Mn, and Zn (AAC) during gestation. Adapted from Harvey et al. [85]. Heifers were weaned on d 367 of the experiment, at ~7 mo of age. A treatment × day interaction was detected (*p* < 0.01). Within days: † 0.05 ≤ *p* ≤ 0.10; * *p* ≤ 0.05; ** *p* < 0.01.

In Harvey et al. [85], heifers born to AAC-supplemented dams reached puberty earlier in the experiment compared with INR cohorts (Figure 1). Puberty onset is highly influenced by body composition and development [86], whereas attainment and age at puberty differed between heifers from AAC and INR cows despite similar BW gain between treatments (Table 5). In turn, heifers from INR cows had greater mRNA expression of *myogenin* and tended to have greater mRNA expression of paired box gene 7 (PAX7) in the LM compared with AAC cohorts (Table 5). *Myogenin* is a regulatory factor in the LM muscle that influences postnatal growth through differentiation and fusion of satellite cells with existing muscle fibers [5,87], whereas PAX7 is necessary for satellite cell specification and survival [88,89]. Hence, Harvey et al. [85] speculated that heifers born to INR supplemented cows had a greater population of satellite cells undergoing differentiation at the time of sampling. Further, no treatment differences were detected in the LM for genes associated with adipogenic activities (Table 5), however, the intramuscular region is typically the last depot for adipose tissue to be deposited in the growing animal [90] and the puberty process is more likely influenced by subcutaneous fat accretion [91,92,93]. Myogenic factors are downregulated as cattle mature and muscle fibers are fully developed [5,94]; therefore Harvey et al. [85] noted that treatment differences noted in the LM for mRNA expression of *myogenin* and PAX7 may be indicative of accelerated physiological maturation in heifers born to AAC-supplemented cows [95].

The role of trace mineral source on reproductive performance is complex, especially given the plethora of processes each element participates in, and the essentiality of each in all aspects of development [32]. Previous research demonstrated lactating beef cows supplemented with organic Co, Cu, Mn, and Zn yielded a greater number of culturable oocytes and transferable embryos compared with cohorts receiving sulfate sources of the same elements [47]. Primordial germs cells within the developing ovary actively utilize machinery and enzymes against reactive oxygen species (ROS) to maintain cell integrity [96], and increased antioxidant enzyme activity and decreased ROS production during germ cell development results with trace mineral supplementation [97,98]. Moreover, trace mineral deficiency, specifically Zn, leads to epigenetic defects and impaired ovarian development during the fetal period [99]. Thus, it became plausible to speculate that supplementing AAC to gestating cows favored the ovarian development of heifer offspring, protecting ovarian cells and follicles from endogenous ROS during development compared to heifers born to INR-supplemented cows [85].

## 5. Conclusions

Nutritional manipulation during periods of developmental plasticity such as the embryonic, fetal, and neonatal periods exert lasting effects on skeletal muscle and adipose tissue development, health, and overall performance of offspring [3]. Identifying specific nutritional strategies targeting these periods of development provides a unique opportunity to optimize the efficiency and profitability of beef production systems. Supplementing beef cows with organic complexed sources of Co, Cu, Mn, and Zn during gestation instead of no supplementation, enhanced passage of Zn and Cu from maternal to fetal tissues, resulting in life-long programming effects on offspring productivity and health. Further, heifers born to cows receiving organic-complexed trace minerals exhibited hastened puberty attainment compared with cohorts from cows that received sulfate sources. These outcomes were not associated with trace mineral deficiencies in either scenario, but rather the programmatic effects of additional Cu, Co, Mn, and Zn intake by cows. Results from these experiments are novel, and suggest that supplementing organic-complexed Co, Cu, Mn, and Zn to gestating beef cows enhances offspring growth and development, leading to long-term effects on performance, health, and accelerated reproductive development of female progeny.

## Figures and Tables

**Table 1:** Physiological, health, and productive responses from beef cows (and their offspring) that received diets containing no supplemental Cu, Co, Mn, and Zn (CON); sulfate sources of Cu, Co, Mn, and Zn (INR); or organic complexed source of Cu, Mn, Co, and Zn (AAC) during the last trimester of gestation ^1^.

Item	CON	INR	AAC
Cow liver ^2^, mg/kg			
Co	0.21 ^a^	0.40 ^b^	0.44 ^c^
Cu	69 ^a^	155 ^b^	129 ^c^
Mn	8.7	9.0	8.7
Zn	211 ^a^	230 ^b^	235 ^b^
Cotyledon ^3^, mg/kg			
Co	0.13 ^a^	0.20 ^b^	0.24 ^b^
Cu	3.88 ^a^	4.75 ^a,b^	5.12 ^b^
Mn	22.0	18.2	22.9
Zn	65	66	68
Calf liver ^3^, mg/kg			
Co	0.09 ^a^	0.12 ^b^	0.13 ^b^
Cu	362 ^a^	428 ^a,b^	450 ^b^
Mn	5.82	5.22	5.83
Zn	456 ^a^	562 ^a,b^	660 ^b^
Calf birth BW, kg	42.1	41.6	40.8
Calf weaning BW, kg	212 ^a^	223 ^a,b^	236 ^b^
Treated for BRD symptoms ^4^, %	42.3 ^a^	59.1 ^a^	20.0 ^b^
BW prior to slaughter, kg	649 ^a^	663 ^a,b^	680 ^b^
Hot carcass weight, kg	409 ^a^	418 ^a,b^	428 ^b^

^1^ Adapted from Marques et al. [59], and means within row with different superscripts (^a,b,c^) differ (*p* ≤ 0.05). ^2^ Samples collected via needle biopsy 2 weeks prior to the beginning of the calving season. ^3^ Samples collected within 3 h of calving via needle biopsy. ^4^ BRD = bovine respiratory disease. Treated for BRD symptoms in the growing lot.

**Table 2:** Concentrations of Co, Cu, Mn, and Zn and expression of genes in the liver in beef cows that received diets containing supplemental sulfate sources of Co, Cu, Mn, and Zn (INR) or organic-complexed source of Co, Cu, Mn, and Zn (AAC) during gestation ^1,2^.

Item	INR	AAC
Liver mineral profile		
Co, mg/kg		
Late gestation	0.59 ^b^	0.68 ^a^
Calving	0.58	0.55
Cu, mg/kg		
Late gestation	125 ^a^	81.9 ^b^
Calving	118 ^a^	42.9 ^b^
Mn, mg/kg		
Late gestation	10.5	10.0
Calving	12.0	10.6
Zn, mg/kg		
Late gestation	307	341
Calving	173 ^a^	129 ^b^
Gene mRNA expression ^3^		
*Cu-transporter protein*		
Late gestation	1.67	1.70
Calving	1.95	1.75
*Metallothionein 1A*		
Late gestation	26.1	22.8
Calving	36.4 ^b^	65.6 ^a^
*Superoxide dismutase 1*		
Late gestation	1.99	2.11
Calving	2.02	2.04

^1^ Adapted from Harvey et al. [72], and means within row with different superscripts (^a,b^) differ (*p* ≤ 0.05). ^2^ Liver samples were collected via needle biopsy during late gestation and upon calving. ^3^ Values are expressed as relative fold change compared with threshold cycle of reference genes analyzed within the same sample.

**Table 3:** Concentrations of Co, Cu, Mn, and Zn and expression of genes in the liver in beef cows that received diets containing supplemental sulfate sources of Co, Cu, Mn, and Zn (INR) or organic-complexed source of Co, Cu, Mn, and Zn (AAC) during gestation ^1,2^.

Item	INR	AAC
Liver mineral profile		
Co, mg/kg		
Cotyledon	0.494	0.533
Calf birth	0.202	0.188
Calf 24 h after birth	0.153	0.148
Cu, mg/kg		
Cotyledon	8.38	9.07
Calf birth	394	399
Calf 24 h after birth	303	291
Mn, mg/kg		
Cotyledon	18.9	18.4
Calf birth	5.96	6.06
Calf 24 h after birth	4.70	4.83
Zn, mg/kg		
Cotyledon	90.9	93.4
Calf birth	823	869
Calf 24 h after birth	676	637
Gene mRNA expression ^3^		
*Cu-transporter protein*		
Birth	2.19	2.23
24 h after birth	2.51	2.53
*Metallothionein 1A*		
Birth	33.7	32.9
24 h after birth	59.4	68.4
*Superoxide dismutase 1*		
Birth	2.92	2.96
24 h after birth	2.77	2.70

^1^ Adapted from Harvey et al. [72], and no treatment differences were detected (*p* ≥ 0.38). ^2^ Cotyledon and calf liver samples (needle biopsy) were collected at birth and again 24 h after birth. ^3^ Values are expressed as relative fold change compared with threshold cycle of reference genes analyzed within the same sample.

**Table 4:** Lactation responses of beef cows that received diets containing supplemental sulfate sources of Co, Cu, Mn, and Zn (INR) or organic-complexed Co, Cu, Mn, and Zn (AAC) during gestation, and performances responses of their offspring until weaning ^1^.

Item	INR	AAC
Lactation responses		
Milk yield, kg/d	6.62	6.98
Milk mineral profile		
Co, ppm		
Colostrum	0.0052	0.0055
Milk	0.00030	0.00035
Cu, ppm		
Colostrum	0.985	0.997
Milk	0.049	0.062
Mn, ppm		
Colostrum	0.061	0.055
Milk	0.014	0.016
Zn, ppm		
Colostrum	15.3	13.7
Milk	0.014	0.016
Calf performance responses		
Calf birth BW, kg	30.5	30.8
Calf weaning BW, kg	183	178

^1^ Adapted from Harvey et al. [72], and no treatment differences were detected (*p* ≥ 0.19).

**Table 5:** Productive responses and mRNA expression of *longissimus* muscle genes in replacement heifers born from beef cows that received diets containing supplemental sulfate sources of Co, Cu, Mn, and Zn (INR) or organic complexed source of Co, Cu, Mn, and Zn (AAC) during gestation ^1^.

Item	INR	AAC
Initial body weight ^2^, kg	202	197
Final body weight ^2^, kg	332	326
Average daily gain ^2^, kg/d	0.618	0.604
Final puberty attainment ^3^, %	83.5	86.4
Age at puberty, d	418 ^a^	399 ^b^
Body weight at puberty, kg	319	310
mRNA expression ^4^		
*Adipocyte fatty acid binding protein*	4.68	5.10
*Myogenin*	4.59 ^a^	2.87 ^b^
*Paired box gene 7*	1.91 ^x^	1.70 ^y^
*Peroxisome proliferator-activated receptor-γ*	1.62	1.53

^1^ Adapted from Harvey et al. [85], and means within row with different superscripts differ at *p* ≤ 0.05 (^a,b^) or *p* = 0.09 (^x,y^). ^2^ Heifer body weight (BW) were obtained 45 d after weaning (initial) and at the end of the experiment (210 d later), which were used to calculate average daily gain. ^3^ Evaluated according to plasma progesterone concentrations in samples collected weekly. ^4^ Values are expressed as relative fold change compared with threshold cycle of reference genes analyzed within the same sample.

## Data Availability

Not applicable.
